# Identification and coregulation pattern analysis of long noncoding RNAs in the mouse brain after *Angiostrongylus cantonensis* infection

**DOI:** 10.1186/s13071-024-06278-6

**Published:** 2024-05-07

**Authors:** Dong-Hui Cheng, Tian-Ge Jiang, Wen-Bo Zeng, Tian-Mei Li, Yi-Dan Jing, Zhong-Qiu Li, Yun-Hai Guo, Yi Zhang

**Affiliations:** 1 National Institute of Parasitic Diseases, Chinese Center for Disease Control and Prevention (National Center for Tropical Diseases Research); Key Laboratory of Parasite and Vector Biology, National Health Commission; National Key Laboratory of Intelligent Tracking and Forecasting for Infectious Diseases; WHO Collaborating Centre for Tropical Diseases, National Center for International Research On Tropical Diseases, Shanghai, People’s Republic of China; 2 School of Global Health, National Center for Tropical Disease Research, Shanghai Jiao Tong University, Shanghai, People’s Republic of China; 3 School of Life Sciences, Fudan University, Shanghai, People’s Republic of China; 4 Dali Prefectural Institute of Research and Control On Schistosomiasis, Yunnan, People’s Republic of China

**Keywords:** *Angiostrongylus cantonensis*, Long noncoding RNA, RNA-Seq, Angiostrongyliasis, Parasitic disease

## Abstract

**Background:**

Angiostrongyliasis is a highly dangerous infectious disease. *Angiostrongylus cantonensis* larvae migrate to the mouse brain and cause symptoms, such as brain swelling and bleeding. Noncoding RNAs (ncRNAs) are novel targets for the control of parasitic infections. However, the role of these molecules in *A. cantonensis* infection has not been fully clarified.

**Methods:**

In total, 32 BALB/c mice were randomly divided into four groups, and the infection groups were inoculated with 40 *A. cantonensis* larvae by gavage. Hematoxylin and eosin (H&E) staining and RNA library construction were performed on brain tissues from infected mice. Differential expression of long noncoding RNAs (lncRNAs) and mRNAs in brain tissues was identified by high-throughput sequencing. The pathways and functions of the differentially expressed lncRNAs were determined by Kyoto Encyclopedia of Genes and Genomes (KEGG) and Gene Ontology (GO) analyses. The functions of the differentially expressed lncRNAs were further characterized by lncRNA‒microRNA (miRNA) target interactions. The potential host lncRNAs involved in larval infection of the brain were validated by quantitative real-time polymerase chain reaction (qRT‒PCR).

**Results:**

The pathological results showed that the degree of brain tissue damage increased with the duration of infection. The transcriptome results showed that 859 lncRNAs and 1895 mRNAs were differentially expressed compared with those in the control group, and several lncRNAs were highly expressed in the middle–late stages of mouse infection. GO and KEGG pathway analyses revealed that the differentially expressed target genes were enriched mainly in immune system processes and inflammatory response, among others, and several potential regulatory networks were constructed.

**Conclusions:**

This study revealed the expression profiles of lncRNAs in the brains of mice after infection with *A. cantonensis*. The lncRNAs H19, F630028O10Rik, Lockd, AI662270, AU020206, and Mexis were shown to play important roles in the infection of mice with *A. cantonensis* infection.

**Graphical Abstract:**

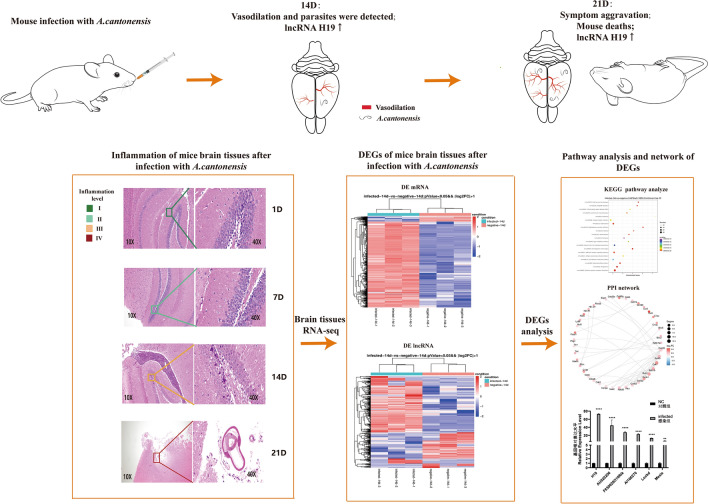

**Supplementary Information:**

The online version contains supplementary material available at 10.1186/s13071-024-06278-6.

## Background

Parasitic diseases, especially helminth infections, which are among the most important neglected tropical diseases (NTDs), pose a serious threat to humans globally [1]. More than 2 billion people worldwide are infected with helminths, resulting in more than 12 million disability-adjusted life years (DALYS) [2, 3].

For example, the global prevalence of *Ascariasis* is reported to be 446 million infections, hookworms cause 173 million infections, schistosomiasis affects more than 200 million people in 74 countries, and the World Health Organization estimates that globally, more than 600 million people are infected by *Strongyloides stercoralis* [4–6]. *Angiostrongylus cantonensis* is a zoonotic parasitic nematode with a complex indirect life cycle [7, 8] and is one of the most common pathogens causing eosinophilic meningoencephalitis [9]. The third-stage larvae of *A. cantonensis* are its infective form [10], and its life cycle is completed mainly in rats and snails; gastropods, such as slugs and snails, are its intermediate hosts and mammals, such as rats, are its final hosts [11]. More than 20 vertebrate species, including humans, may serve as accidental hosts, while amphibians and fish may serve as transfer hosts [12–14]. *A. cantonensis* may develop into adult nematodes in rats only; mice and humans are nonpermissive hosts [15]. *A. cantonensis* cannot mature into worms in mice. Humans are mainly infected by raw or semiraw consumption of pathogen-carrying fruits and vegetables and undercooked intermediate hosts [16]. Early symptoms of *A. cantonensis* infection includes nausea, vomiting, breathing difficulties, headaches, and low-grade fever. Later, the infection progresses to severe chronic headache, paralysis, and even coma or death [17].

The disease is prevalent mainly in Southeast Asia, the Caribbean, the Pacific basin, and other regions [18]. In recent years, the global number of cases of infection with *A. cantonensis* has increased annually [9]. The disease burden of *A. cantonensis* is likely underestimated [11]. In China, the first case of human infection with *A. cantonensis* was reported in Taiwan in 1944 when *A. cantonensis* was found in the cerebrospinal fluid of a young man exhibiting meningeal symptoms and eosinophils in the cerebrospinal fluid [19]. From 1945 to 2008, a total of 769 cases were reported in China, accounting for approximately 27.22% of the global number of cases and posing a serious threat to people’s health [16].

Noncoding RNAs (ncRNAs) are RNAs that do not encode proteins and account for approximately 98% of the human genome [20, 21]. NcRNAs include ribosomal RNA (rRNA), transfer RNA (tRNA), long noncoding RNA (lncRNA), circular RNA (circRNA), microRNA (miRNA), etc.; lncRNAs are conserved RNAs with a length of > 200 nucleotides [22, 23]. With the flourishing development of high-throughput technologies, the role of lncRNAs in the growth and development of living organisms as well as in disease processes has gradually been revealed. LncRNAs are key genetic regulators of different biological processes and are involved in regulating epigenetic regulation, cell differentiation, the cell cycle, and immune response [24]. An increasing number of studies have shown that lncRNAs can act as competitive endogenous RNAs that bind to miRNAs and participate in various biological processes [25, 26].

A growing emphasis has been placed on zoonotic diseases with the idea of “One Health” [27], of which *A. cantonensis* is one of the most important emerging diseases. At present, the main treatment for angiostrongyliasis is the use of anthelmintic drugs, which can relieve symptoms and reduce disease duration. However, this treatment may also lead to the release of intracellular contents from dying worms to increase the inflammatory response. Moreover, lncRNAs show promise as novel biomarkers and therapeutic targets for various diseases [28]. Consequently, for better prevention and treatment of angiostrongyliasis and to interrupt transmission of the disease, this study used a BALB/c mouse model to mimic a human infection model to screen differentially expressed lncRNAs in the brain tissue of infected mice and validate their dynamic changes during the course of the infection. This study will provide therapeutic targets and new diagnostic protocols for the treatment of angiostrongyliasis. In addition, the functions of the differentially expressed lncRNAs were further identified by lncRNA‒miRNA target interactions.

## Methods

### Mouse model establishment and hematoxylin and eosin (H&E) staining

*Achatina fulica* were originally obtained from Kaiping, Guangdong, China, and the lung tissues were dissected, isolated, and ground. The third-stage larvae of *A. cantonensis* were removed from the ground homogenate. Thereafter, 32 6–8 week-old BALB/c female rats (Shanghai Jihui Co., Ltd.) were randomly divided into a negative control group and an infected group at a ratio of 3:5. Each mouse in the experimental group was gavaged with 40 third-stage larvae, and the mice in the negative control group were gavaged with saline. After larval infection, brain tissues were collected at 1, 7, 14, and 21 days, fixed in 4% paraformaldehyde, embedded in paraffin, dewaxed in xylene, immersed in different concentrations of ethanol and stained with H&E. Pathological changes in the brains of infected mice at different infection times were observed through H&E staining. After H&E staining, the nuclei were stained blue, and the cytoplasm was stained pink [29].

### Ribonucleic acid extraction library construction

Total RNA was extracted from mouse brain tissue samples at 14 days after infection using TRIzol reagent, RNA purity was assessed, RNA quantification was performed using a NanoDrop 2000 spectrophotometer (Thermo Scientific, USA), and RNA integrity was assessed using an Agilent 2100 Bioanalyzer (Agilent Technologies, Santa Clara, CA, USA). Samples that passed quality control were used for subsequent library construction. Ribosomal RNA was removed using the Ribo-off rRNA Depletion Kit (Vazyme, Nanjing, China). The transcriptome library was constructed using the VAHTS Universal V6 RNA-seq Library Prep Kit according to the instructions. The whole transcriptome was sequenced and analyzed by Shanghai Ouyi Biotechnology Co.

### RNA sequencing and differentially expressed gene analysis

The libraries were sequenced using the Illumina NovaSeq 6000 sequencing platform, and 150 bp bipartite reads were generated. Approximately 125,994 M raw reads were obtained from each sample. Raw reads in fastq format were processed using fastp software, and clean reads were obtained by removing low-quality reads for subsequent data analysis [30]. Negative control group comparisons were performed using HISAT2 software [31]. The read counts for each gene were obtained by HTSeq-count [32], and gene expression (FPKM) calculations were performed to select differentially expressed genes [33].

Differentially expressed gene analysis was performed using DESeq2 software, where genes that met the thresholds of *q* value (adjusted *P* value) < 0.05 and fold change > 2 or fold change < 0.5 were defined as differentially expressed genes (DEGs) [34]. Hierarchical clustering analysis of DEGs was performed using R (v 3.2.0) to demonstrate the expression patterns of genes across samples and groups. Subsequently, Gene Ontology (GO) and Kyoto Gene and Genome Encyclopedia (KEGG) pathway enrichment analyses of DEGs based on hypergeometric distribution algorithms were used to screen for significantly enriched functional entries [35, 36].

### Quantitative real-time PCR (qPCR) validation

In total, six DE lncRNAs were selected for qRT‒PCR analyses to validate the DEG‒Seq results and their dynamics during infection. Reverse transcription was performed using a reverse transcription system kit (TaKaRa, Japan). The primer sequences are shown in Additional file 1, with GAPDH serving as the internal reference primer. Quantitative real-time PCR (qRT‒PCR) analysis was performed using SYBR® Green Real-Time Fluorescent Quantitative PCR Premix (TaKaRa, Japan).

### Protein‒protein interaction (PPI) network construction

The STRING database (http://string-db.org/) was used to predict interactions between proteins, and a combined PPI score greater than 0.4 for differentially expressed mRNAs was used as a critical value [29]. Network mapping of the relationships of the top 50 DE mRNAs was performed on the basis of interaction score sorting.

### LncRNA–mRNA interaction study

The correlation between the six samples in the infected group and the negative control group was calculated using the Pearson correlation test. The correlation analysis set a threshold of an absolute value of the correlation coefficient greater than or equal to 0.80 and a *P* value less than or equal to 0.05. Differentially expressed lncRNAs and genes from the same differential comparison group were identified using Circos plotting software [37].

A hypergeometric distribution test was utilized to identify the miRNAs with the greatest impact among the differentially expressed lncRNAs. For the total differential lncRNA enrichment results, the top 300 miRNA‒lncRNA interaction pairs with smaller *P* values were selected in order of *P* value, and the R network package was used to map the lncRNA‒miRNA targets [38].

### Statistical analysis

Statistical analysis was performed using GraphPad Prism ver. 8.0.2 (GraphPad Software, Inc., San Diego, CA, USA). The expression level of each gene was represented as an FC according to the 2^–△△Ct^ method. The Student’s *t* test was used to analyze the differences between the groups. All the data are expressed as the mean ± standard deviation. All experiments were performed on no fewer than three biological replicates. Significance was defined as a *P* value < 0.05 [39].

## Results

### Animal model construction and pathological changes in brain tissue

**Fig. 1:**
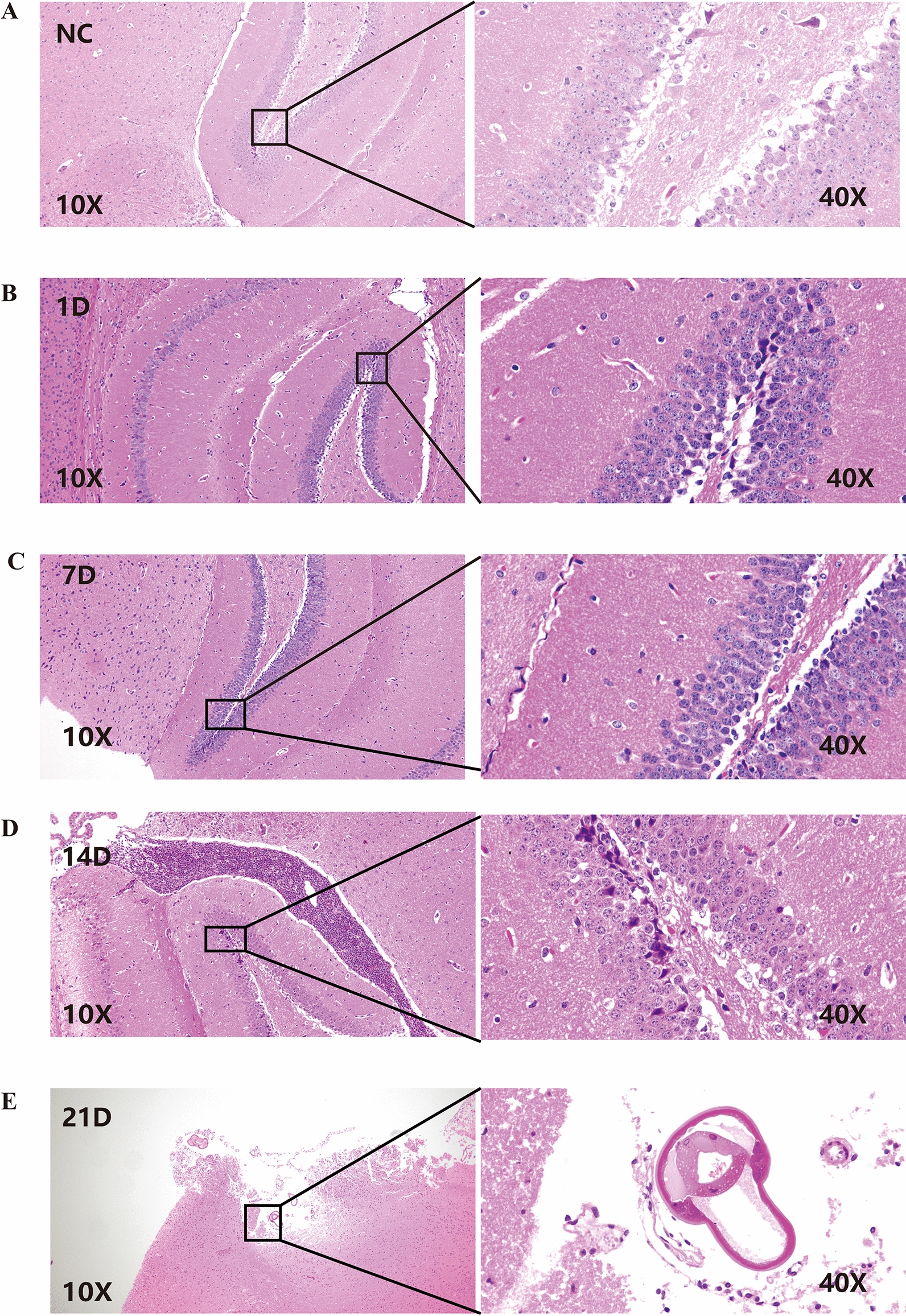
Hematoxylin and eosin staining of mouse brain tissue samples. Negative control group (**A**), 1 day after infection (**B**), 7 days after infection (**C**), 14 days after infection (**D**), and 21 days after infection (**E**)

*A. cantonensis* larvae were found in the brains of mice 14 days after *A. cantonensis* infection. These findings confirmed the successful establishment of an animal model of *A. cantonensis* infection [40]. For the mouse infection model, no obviously abnormal brain tissue was observed at 1 day or 7 days. After 14 days of infection, a few cone cells with degeneration and cytoplasmic consolidation were occasionally observed in the hippocampus, with some degree of damage, and increased inflammatory cell infiltration was observed around the brain tissue and in the ventricles; eosinophils were also observed. These symptoms were significantly exacerbated on the 21st day postinfection, and parasites were detected in the brain tissue (Fig. 1).

### Identification of differentially expressed lncRNAs and mRNAs

After quality trimming, an average of 104.088 M clean reads were obtained for each sample. The sequencing quality data are provided in Additional file 1 (Additional file 2: Table S2). The mean Q30 score was 94.34% (i.e., the probability of a correct base call was 94.34%), demonstrating the good quality of the RNA-sequencing (RNA-seq) data.

**Fig. 2:**
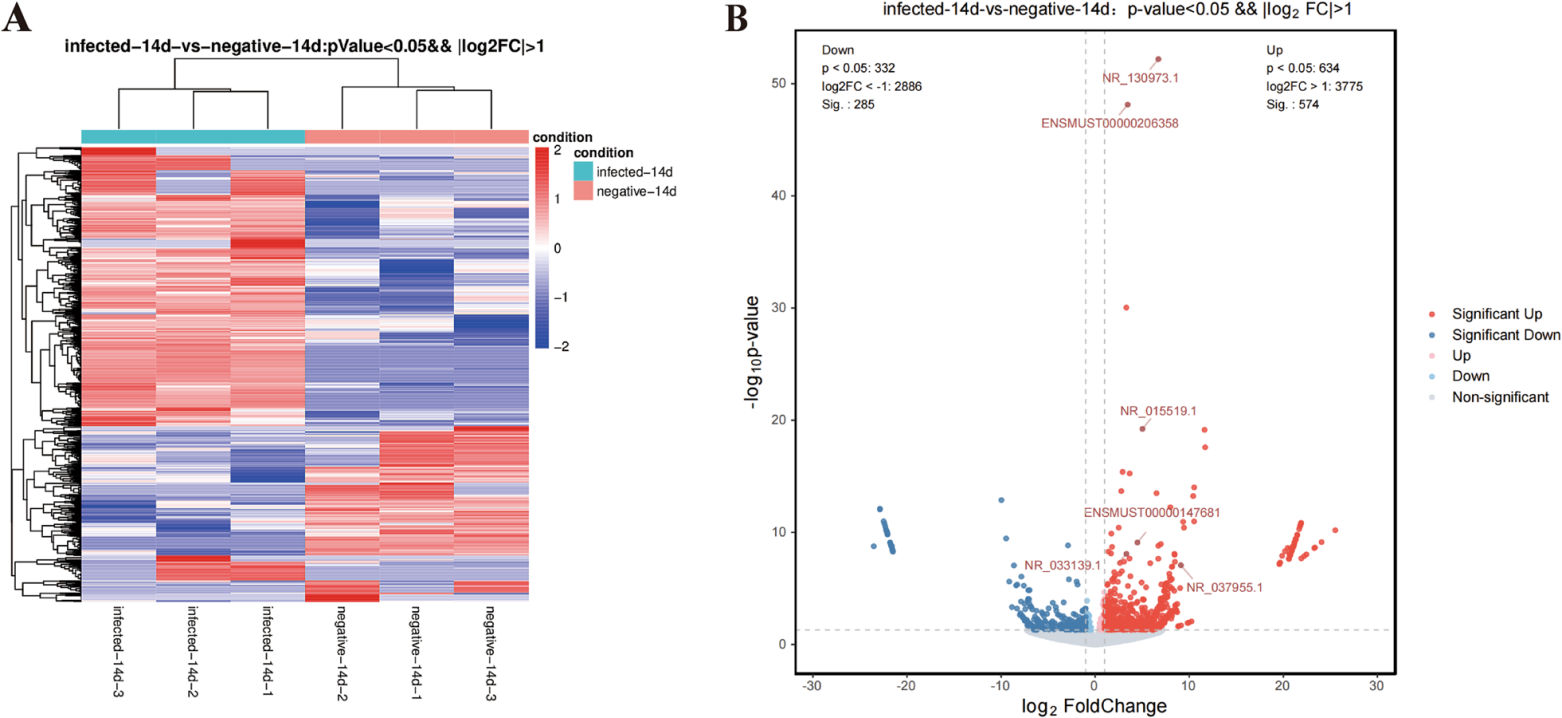
Differentially expressed lncRNA profiles. Differential lncRNA grouping clustering graph (**A**): the graph indicates relatively high expression lncRNA in red and relatively low expression lncRNA in blue. Differential expression volcano graph (**B**): the differences resulting from the comparison are reflected in the volcano graph, with nonsignificantly different lncRNA in gray and significantly different lncRNA in red and green; the horizontal axis is log2FoldChange, and the vertical axis is −log10 *P* value

**Fig. 3:**
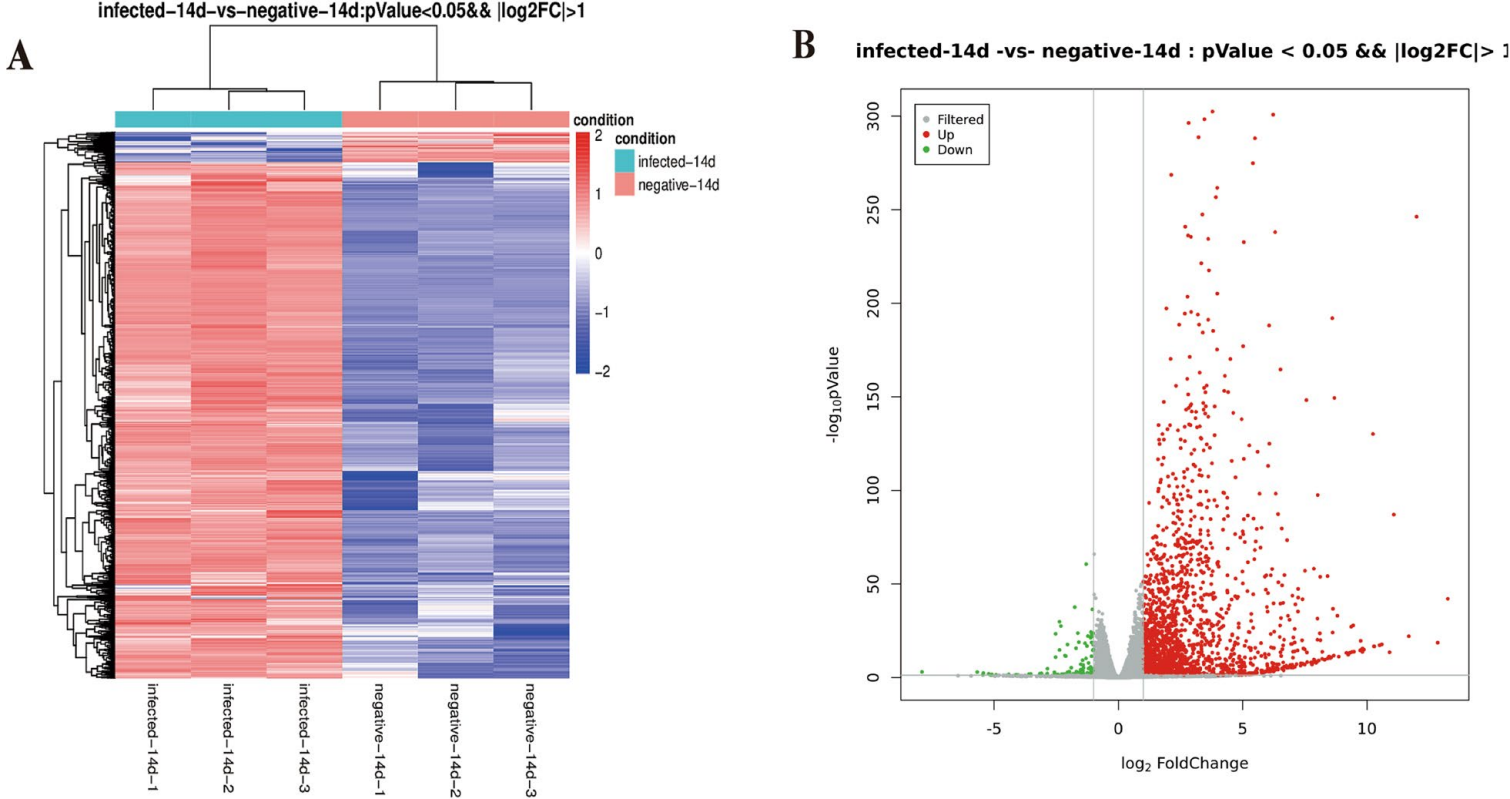
Differentially expressed mRNA profiles. Differential mRNA grouping clustering graph (**A**): the graph indicates relatively high expression mRNAs in red and relatively low expression mRNAs in blue. Differential expression volcano graph (**B**): the differences resulting from the comparison are reflected in the volcano graph, with nonsignificantly different mRNAs in gray and significantly different mRNAs in red and green; the horizontal axis is log2FoldChange, and the vertical axis is −log10 *P *Value

The sequencing data revealed a total of 859 differentially expressed lncRNAs (differences > 2, *q* < 0.05), 574 of which were upregulated and 285 of which were downregulated (Fig. 2A, B Additional file 3: Table S3). In addition, 1895 differentially expressed mRNAs (difference in ploidy > 2, *q* < 0.05) were screened, of which 1790 mRNAs were upregulated and 105 mRNAs were downregulated (Fig. 3A, B, Additional file 4: Table S4).

### GO analysis and KEGG pathway analysis

The expression of many lncRNAs/mRNAs was significantly dysregulated in mice infected with *A. cantonensis* 14 days after infection. To reveal the function of aberrant lncRNAs/mRNAs, we performed GO and KEGG pathway enrichment analyses.

**Fig. 4:**
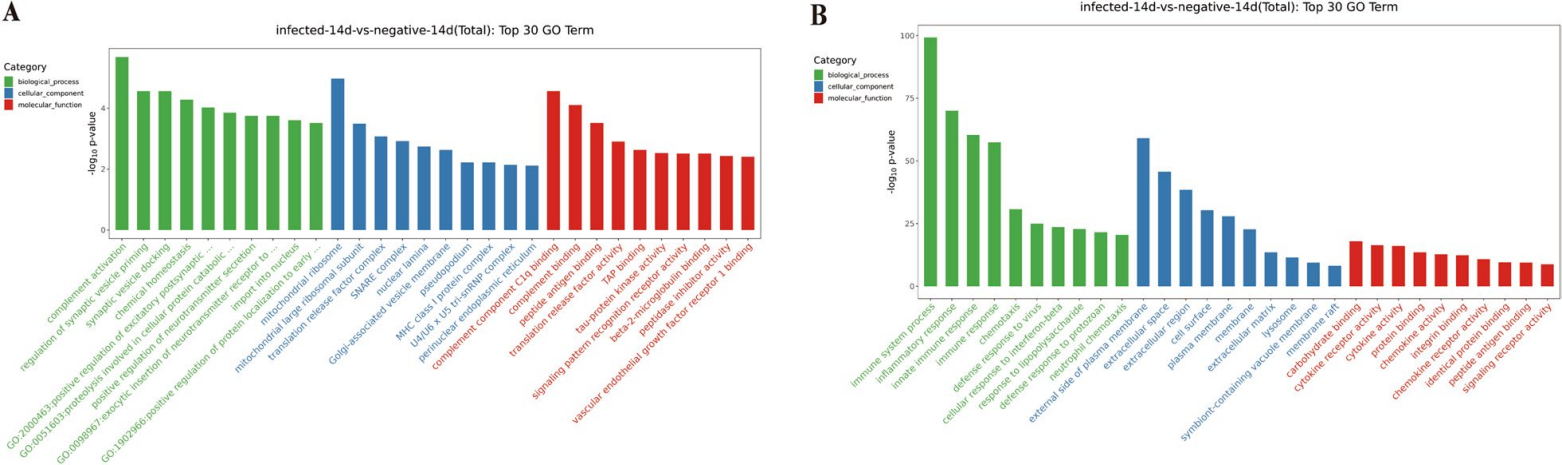
GO enrichment analysis results. The horizontal axis is the GO entry name, and the vertical axis is −log10 *P *value

The top 10 GO terms were sorted by the corresponding −log10p value under each of the three categories in descending order; the top 10 GO terms were plotted as the top 30 GO enrichment analysis results (Additional file 5: Table S5 and Additional file 6: Table S6). GO analysis of the differentially expressed contiguous genes of the lncRNAs revealed (Fig. 4A) that the most significantly enriched biological processes (BPs) were complement activation, regulation of synaptic vesicle initiation, synaptic vesicle docking, chemical in vivo homeostasis, complement binding, and peptide antigen binding. The most significantly enriched cellular components (CCs) were mitochondrial ribosomes, mitochondrial large ribosomal subunits, and translation release factor complexes. The most significant molecular functions (MFs) were complement binding, peptide antigen binding, and translation release factor activity, among others. GO terms of differentially expressed mRNAs (Fig. 4B), including immune system processes, inflammatory response, and involvement in the outer side of the constituent plasma membrane, were significantly enriched.

**Fig. 5:**
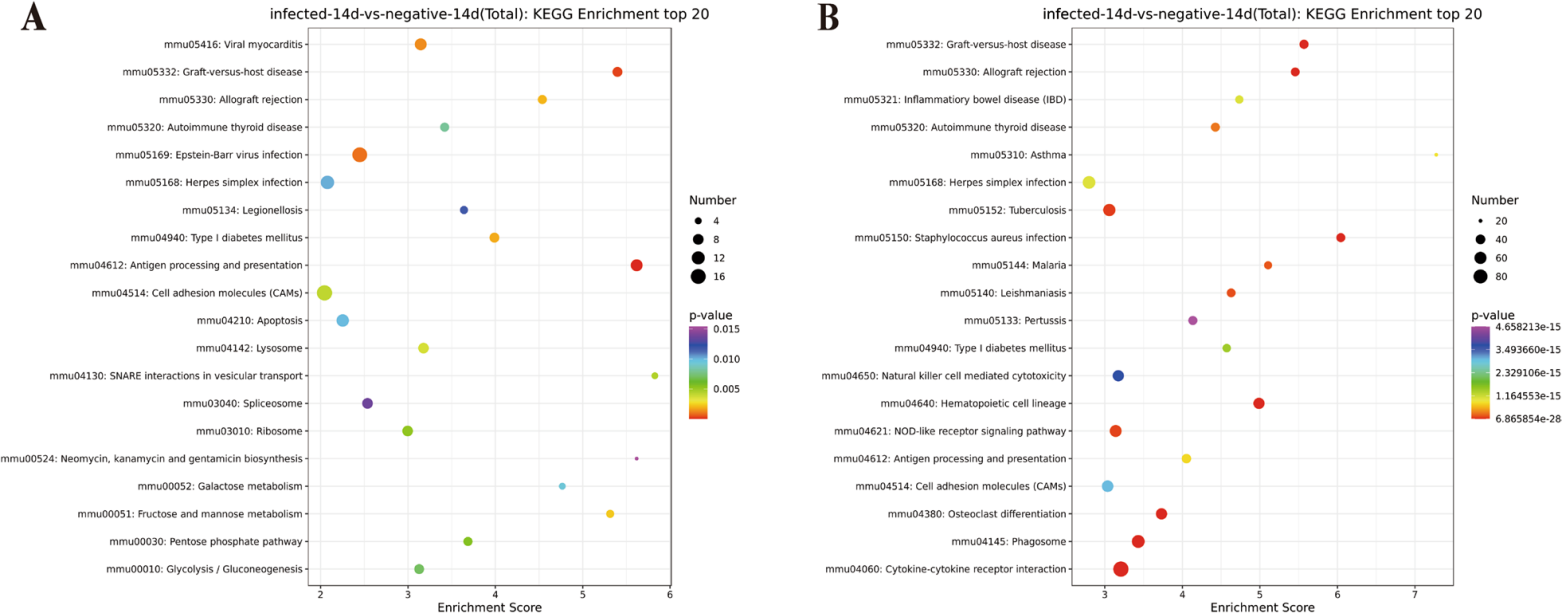
KEGG enrichment top20 bubble map. The horizontal axis enrichment score is the enrichment score, the larger the bubble the more differential genes are contained in the entry, the bubble color changes from purple–blue–green–red; the smaller the enrichment *P* value, the greater the significance

KEGG pathway analysis identified differentially expressed lncRNA genes (Fig. 5A and Additional file 7: Table S7), which were mainly associated with antigen processing and presentation signalling pathways, graft-versus-host disease, and cell adhesion molecules. Analysis of the differentially expressed mRNAs revealed cytokine‒cytokine receptor interactions, the NOD-like receptor signalling pathway and phagosomes (Fig. 5B and Additional file 8: Table S8).

### LncRNA and mRNA coexpression and protein interaction network analysis

**Fig. 6:**
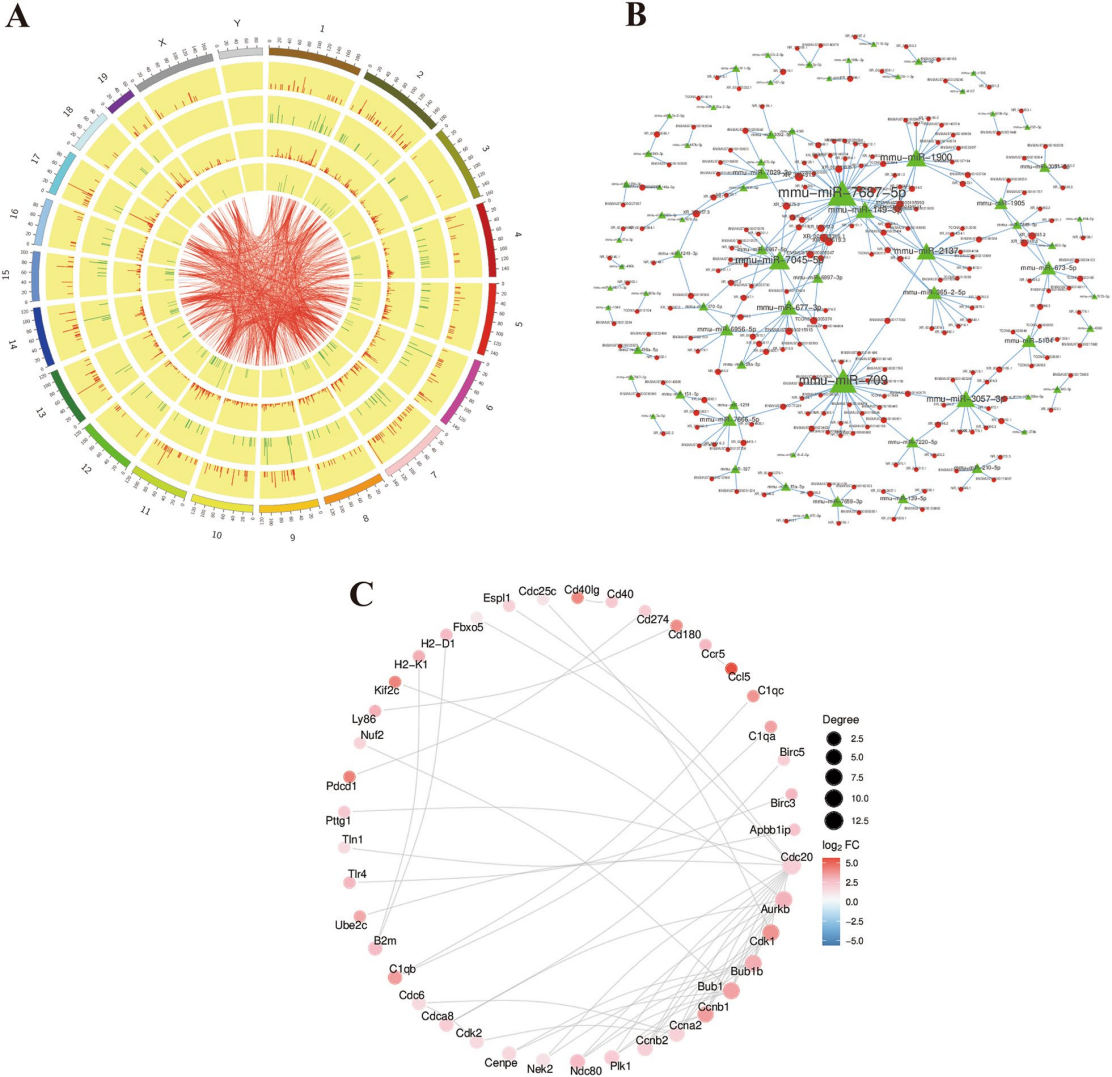
Coexpression circos plot (**A**) The outermost circle is the schematic of the autosomal distribution of the species; the second and third circles are the distribution of differentially expressed genes on the chromosome, red lines indicate upregulation, green lines indicate downregulation. The higher the bar, the higher the number of differential genes in the region; the fourth and fifth circles are the distribution of differentially expressed lncRNA on the chromosome, expressed in the same form as gene. The internal connecting lines indicate the correspondence between the Top500 coexpressed lncRNA and gene. lncRNA–miRNA network diagram (**B**): lncRNA are circles, miRNAs are triangles, larger graphs indicate more nodes connected to them. PPI network diagram (**C**): red indicates upregulated differentially expressed genes and blue indicates downregulated differentially expressed genes; the more associated genes, the larger the gene spots

To explore the relationships between differentially expressed lncRNAs and differentially expressed mRNAs, we performed lncRNA‒mRNA coexpression analyses and constructed lncRNA‒mRNA network maps (Fig. 6A, B, Additional file 9: Table S9 and Additional file 10: Table S10). The PPI network of the top 50 DE genes is shown in the STRING database, and the interaction scores between them were all greater than 0.999 (Fig. 6C and Additional file 11: Table S11).

### Target gene network analysis and qPCR validation

**Fig. 7:**
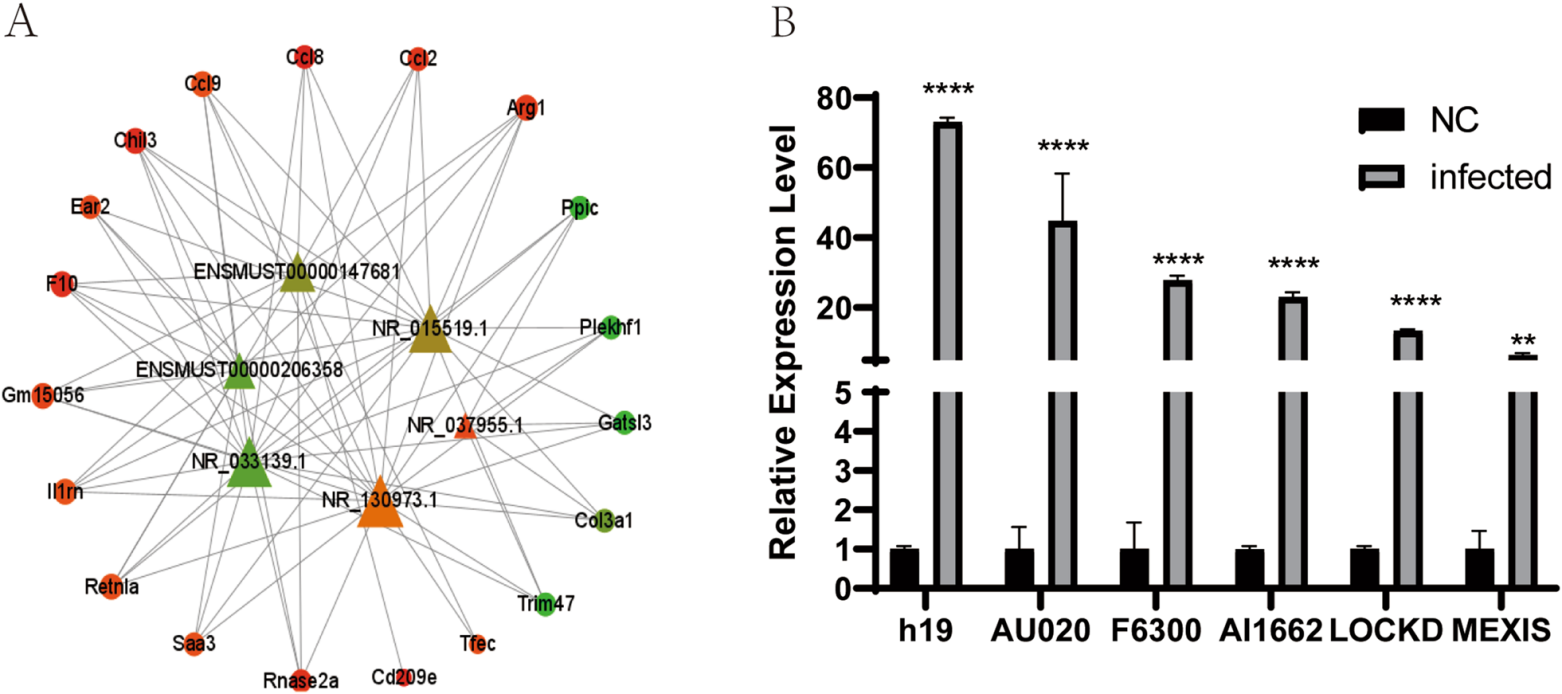
Validation of differential expression of lncRNA and target gene network analysis. The network diagram between lncRNA and target genes (**A**): triangles represent lncRNA and loops represent target genes. The size of the node is determined by the degree of the node, green–yellow–red indicates that log2FC is getting bigger, the darker the color the bigger the difference. qPCR validation graph (**B**): control and test groups were compared by the qPCR method for H19, F630028O10Rik, Lockd, AI662270, AU020206, and Mexis. *P ≤ 0.05, **P ≤ 0.01, ***P ≤ 0.001, and **** *P* ≤ 0.0001

The STRING database was used to predict protein‒protein interactions and map the PPI network of six lncRNAs (Additional file 12: Table S12). The results showed that H19, F630028O10Rik, Lockd, AI662270, AU020206, and Mexis were significantly associated with the target genes (Fig. 7A).

To ensure the accuracy and reliability of the RNA-seq data, we performed RT‒PCR to validate the expression levels of the six lncRNAs. As shown in Fig. 7B, the qPCR results of the six differentially expressed genes were consistent with the sequencing results.

### Dynamic relative expression of DE lncRNAs in the cerebral tissue of mice infected with *A. cantonensis*

**Fig. 8:**
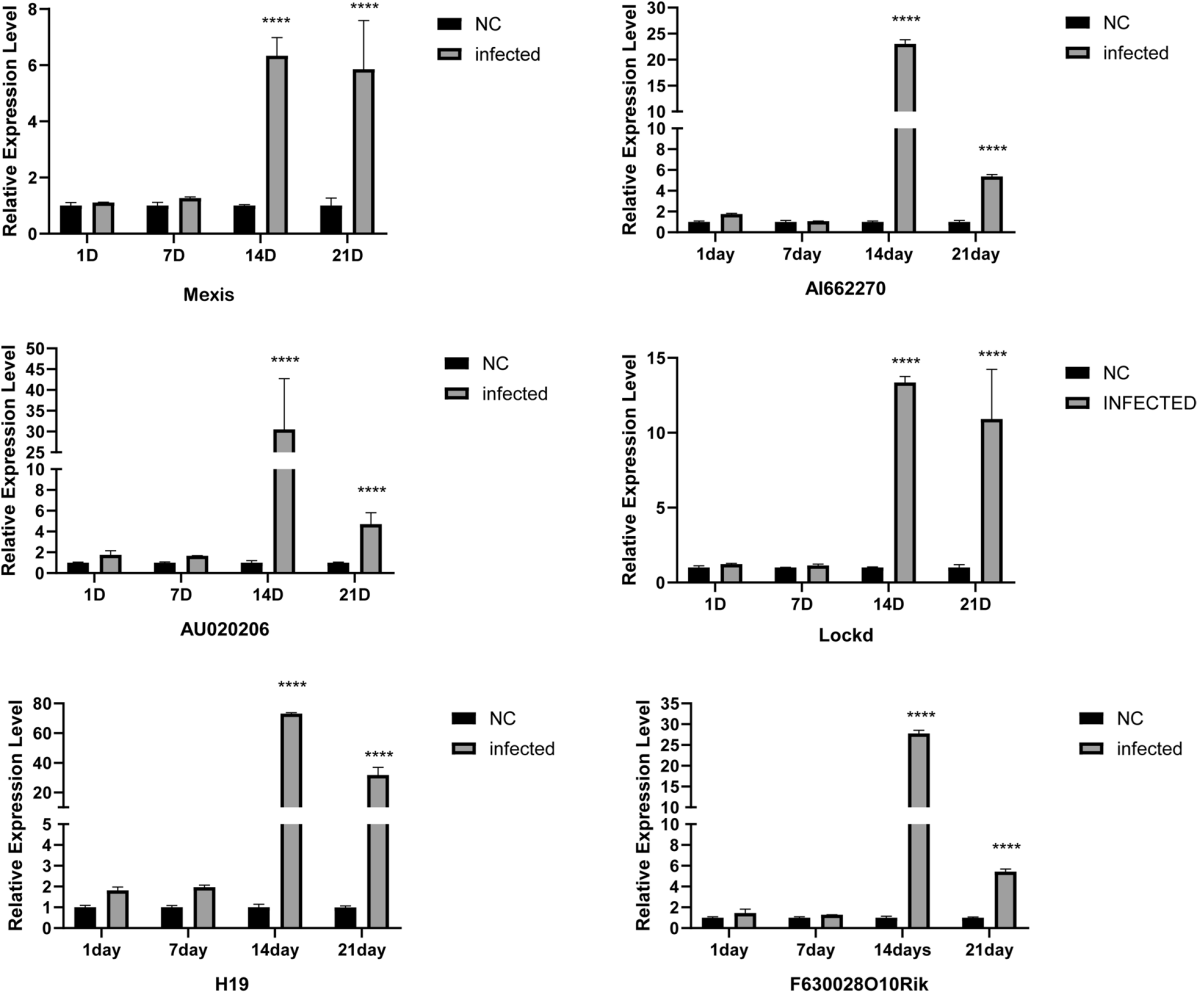
Dynamic relative expression of H19, F630028O10Rik, Lockd, AI662270, AU020206, and Mexis of the cerebrum tissue in the mice model infected with *A. cantonensis.* *P ≤ 0.05, **P ≤ 0.01, ***P ≤ 0.001, and **** *P* ≤ 0.0001

During the period of *A. cantonensis* infection, H19, F630028O10Rik, Lockd, AI662270, AU020206 and Mexis showed dynamic fluctuations in the mouse model compared with the negative control (Fig. 8).

In the models, the expression of these six genes was significantly upregulated at 14 and 21 days after infection with *A. cantonensis* compared with that in the control group at the same age, and the expression level was greatest on day 14. There was no significant difference in the expression of H19, Lockd, or Mexis between 14 and 21 days. The expression levels of the other three genes decreased significantly after 21 days of infection with *A. cantonensis* compared with 14 days.

## Discussion

Angiostrongyliasis is a zoonosis that is widely distributed in more than 30 countries [16]. This condition is often misdiagnosed, and there are no specific drugs available for treating this disease [16]. Therefore, revealing the expression profiles of *A. cantonensis* after infection in an animal model could provide a new theoretical basis for the development of more effective and specific therapeutic methods [41].

In the present study, histopathological analysis of mouse brain tissue showed that with the progression of *A. cantonensis* infection, there were different degrees of damage and worsening of the inflammatory response, characterized by a large number of inflammatory cells infiltrating the brain tissue and ventricles. High-throughput sequencing identified differentially expressed lncRNAs in mouse brain tissues 14 days after *A. cantonensis* infection. A total of 574 lncRNAs were upregulated and 285 lncRNAs were downregulated in the infected group compared with the negative control group. H19, F630028O10Rik, Lockd, AI662270, AU020206, and Mexis were not significantly differentially expressed on days 1 and 7 of *A. cantonensis* infection, but these six lncRNAs were significantly highly expressed on days 14 and 21, and their expression levels peaked on day 14. This result is consistent with the degree of brain tissue injury and associated cytokine and miRNA changes after infection [40, 42]. Moreover, we searched the *Homo sapiens* library using the sequences of these six lncRNAs and found that the lncRNAs Lockd, Mexis, and F630028O10Rik were present in *Homo sapiens*, with similarity rates of 77.78%, 76.39%, and 90.55%, respectively [43, 44]. LncRNAs can orchestrate various physiological processes, and their dysfunction affects a wide range of human diseases [22]. Thus, we speculated that these differentially expressed lncRNAs may also play important roles in the process of infection by *A. cantonensis*.

Previous studies have shown that lncRNA H19 plays an important role in regulating cellular functions in a variety of diseases [45, 46]. H19 can regulate cell proliferation. LncRNA H19 overexpression inhibited mTOR phosphorylation and promoted ULK1 phosphorylation, further promoting cell proliferation, migration, and autophagy [47]. Additionally, H19 can promote tumor cell growth, invasion, and migration through the H19/miR-200a/CDK6/ZEB1 axis [48]. H19 can regulate immunity. In systemic lupus erythematosus (SLE), lncRNA H19 is significantly upregulated and inhibits the proliferation of Treg cells and promotes the conversion of Treg cells to Tfh cells through the direct inhibition of IL-2 production, which results in immune dysregulation and exacerbates autoimmunity [49]. Interleukin-2 cytokine is an important modulator of immune responses [50]. H19 is overexpressed in gastric cancer (GC) cells and contributes to immune escape from GC cells by decreasing immune cell activity and IL-2 expression through the miR-519d-3p/LDHA/lactate axis [51]. In addition, lncRNA H19 can regulate immune cell infiltration through miR-378a-5p/SERPINH1 signaling [52]. The effect of lncRNA H19 on immune regulation is diverse, a recurring theme in literature is the relationship between lncRNA H19 and inflammatory responses [50]. In retinal ischaemia and reperfusion, high H19 expression mediates ceRNET to trigger the mutual activation of NLRP3/NLRP6 inflammatory vesicles, which subsequently initiates GSDMD lysis and microglia scorching [53, 54], leading to a strong proinflammatory response triggered by a substantial release of cellular contents [55]. H19 can regulate inflammatory expression through the NF-κB signalling pathway [56]. H19 can also regulate cell death. H19/miR-21/PDCD4 ceRNET also activates apoptotic cysteine and directly regulates RGC cell apoptosis in retinal I/R injury, exacerbating retinal damage [54]. Moreover, lncRNA H19 upregulation inhibits DUSP5 and activates ERK1/2 to induce autophagic activation, which impairs cell viability, leading to cerebral ischaemia‒reperfusion injury [57]. In the present study, lncRNA H19 was highly expressed in the middle and late stages of infection, which suggests that it may play a role in regulating cell proliferation, apoptosis, and immunity induced by *A. cantonensis* infection, which needs to be further investigated.

Plasmodium infection of LLC tumor-bearing mice inhibited tumor growth and metastasis, and the lncRNA F630028O10Rik was significantly upregulated [58]. Inhibition of lncRNA F630028O10Rik expression promoted the expression of miR-223-3p, resulting in a significant increase in VEGFR2 expression, which promoted tumor angiogenesis and led to tumor expansion [59]. This finding indirectly demonstrated that high F630028O10Rik expression inhibits angiogenesis, which in turn inhibits tumor growth and metastasis. High expression of the lncRNA F630028O10Rik was found in mice with spinal cord injuries, leading to increased expression levels of inflammatory factors and focal death-related genes, among others. Further studies revealed that lncRNA-F630028O10Rik acts as a ceRNA in the miR-1231-5p/Col1a1 axis and enhances microglial scorch death after SCI through activation of the PI3K/AKT pathway [60]. LncRNA F630028O10Rik expression also increased after infection with *A. cantonensis*. Therefore, this molecule may be involved in the regulation of inflammation and apoptosis induced by *A. cantonensis* infection.

Lockd has been experimentally demonstrated to promote myoblast proliferation and acute injury-induced muscle regeneration via the Lockd/DHX36/Anp32e axis [61]. DHX36 as an RNA helicase can regulate the IFN-β signaling pathway by suppressing the formation of the DDX1-DDX21-DHX36 complex and regulating immune homeostasis [62]. DHX36 RNA helicases have been reported to be involved in RLR-mediated type I IFN production after viral infection, and induce an innate immune response [63]. This suggests that Lockd can modulate the immune response induced by the Guangzhou tubular nematode though regulating DHX36 production. In addition, Lockd can positively regulate adjacent Cdkn1b through a cis-acting mechanism and regulate the cell cycle [61, 64]. Lockd was upregulated in this study, suggesting that it may be related to muscle injury and apoptosis caused by the migration of *A. cantonensis* in brain tissue.

In our study, the expression of the lncRNA AI662270 was upregulated. In a previous study, the lncRNA AI662270 was found to affect the molecular properties of M1 kidney cell lines and lead to a reduction in their proliferative capacity [65]. An experiment further revealed that the lncRNA AI662270 can affect the G1 phase of the cell cycle by inducing H3K9me2 in the G1 phase of the cell cycle through the regulation of the lysine methyltransferase G9a [66]. This finding suggested that AI662270 might also inhibit the proliferation of brain tissue cells after infection with *A. cantonensis*.

In addition, both AI662270 and the lncRNA Mexis are highly expressed during the progression of atherosclerosis [67]. AI662270 accelerates the progression of atherosclerosis by directly binding to Abca1 to attenuate Abca1 expression and activity and inhibiting SR-BI expression to promote foam cell formation [68]. Mexis promotes the coactivator action of DDX17, which enhances LXR-mediated Abca1 expression, resulting in increased cholesterol efflux from macrophages, which in turn affects atherosclerotic plaque development [69]. Abca1 is the most abundant protein in inflammatory cells, and the overexpression of Abca1 has antiinflammatory effects [70]. In addition, high Abca1 expression increases the permeability of the blood‒brain barrier [71]. Mice infected with *A. cantonensis* develop blood‒brain barrier impairment and inflammatory responses [72]. This evidence suggests that AI662270 and Mexis may represent breakthroughs in the treatment of angiostrongyliasis. However, persistent high expression of Mexis leads to an inflammatory response [69]. Persistent activation of DDX17 by Mexis-mediated activation of NLRC4 inflammatory vesicles triggers an inflammatory response [73]. Similarly, the lncRNA Mexis was highly expressed in the brain tissue of mice infected with *A. cantonensis*, suggesting that it may play a role in the inflammation caused by *A. cantonensis* infection.

In an atherosclerosis model, lncRNA AU020206 expression was downregulated, while *Bax* and *phosphatidylinositol-4,5-bisphosphate 3-kinase catalytic subunit beta* (*PIK3CB*) gene expression was upregulated, *histone deacetylase 9* (*HDAC9*) expression was upregulated, and the expression of the *CDK4*, *CcNA2*, *CCNE1*, and *CCND3* genes was downregulated [74]. The *Bax* gene is an important apoptotic gene and its high expression triggers apoptosis [74]. *PIK3CB* is involved in apoptosis-related signalling cascades, and its high expression promotes apoptotic cell plaque formation, causing atherosclerosis [75]. *HDAC9*, which is an important gene involved in the regulation of the cell cycle and inflammatory response, is upregulated, *HDAC9* deficiency may promote inflammatory regression, and cyclins and CDKs can form a complex to control cell proliferation [76]. Cyclin-dependent kinases (CDKs) are a family of protein kinases that play a regulatory role in the cell cycle, and cell cycle proteins and CDKs can form complexes to control cell proliferation. Downregulation of CDK4, CcNA2, CCNE1, and CCND3 promoted cell proliferation. Therefore, the lncRNA AU020206 may be involved in cell proliferation, apoptosis and inflammatory responses [74]. The expression of the lncRNA AU020206 was upregulated in mice infected with *A. cantonensis*, suggesting that this molecule is also involved in the regulation of apoptosis and inflammatory responses following *A. cantonensis* infection.

In this study, we only preliminarily explored the changes in the expression of lncRNAs in mice, and the changes in their protein levels were not further investigated. In the future, on the basis of the results of this study, we will use a human cell line model to further study in detail the expression changes and mechanism of action of the relevant genes and proteins after infection with *A. cantonensis*.

## Conclusions

We used RNA-seq to analyze the entire transcriptome profile of mouse brain tissue after *A. cantonensis* infection. Functional predictions via GO and KEGG pathway analyses suggested that these differentially expressed genes play important roles in the infection process of *A. cantonensis*. Our study provides laboratory data to support subsequent studies on the control of angiostrongyliasis.

### Supplementary Information

**Additional file 1:** Table S1. Sequences of the primers used for qPCR validation of RNA-Seq data.

**Additional file 2:** Table S2. Sequencing quality data.

**Additional file 3:** Table S3. The differentially expressed lncRNA.

**Additional file 4:** Table S4. The differentially expressed mRNAs.

**Additional file 5:** Table S5. Gene Ontology (GO) terms of cis target genes of the differentially expressed lncRNA.

**Additional file 6:** Table S6. Gene Ontology (GO) terms of the differentially expressed mRNAs.

**Additional file 7:** Table S7. The KEGG pathways of cis target genes of the differentially expressed lncRNA transcripts.

**Additional file 8:** Table S8. The KEGG pathways of the differentially expressed mRNAs.

**Additional file 9:** Table S9. Differential lncRNA and mRNA co-expression data.

**Additional file 10:** Table S10. LncRNA-miRNA target interaction data.

**Additional file 11:** Table S11. PPI network diagram data.

**Additional file 12:** Network diagram data between lncRNA and target genes.

## Data Availability

The datasets supporting the findings of this article are included within the paper and its supplementary materials. The RNA-seq raw data described in the present study has been submitted to the NCBI Short Read Archive database (https://www.ncbi.nlm.nih.gov/sra) under the bio-project number PRJNA1020087.

## Abbreviations

lncRNA: Long noncoding RNA
ncRNAs: Noncoding RNAs
*A. cantonensis*: *Angiostrongylus cantonensis*
KEGG: Kyoto Gene and Genome Encyclopedia
GO: Gene Ontology
DEGs: Differentially expressed genes
DE lncRNA: Differentially expressed lncRNA
DE mRNAs: Differentially expressed mRNAs
RNA-seq: RNA sequencing
qRT-PCR: Quantitative real-time PCR
